# Modelling climate change impact on the spatial distribution of fresh water snails hosting trematodes in Zimbabwe

**DOI:** 10.1186/s13071-014-0536-0

**Published:** 2014-12-12

**Authors:** Ulrik B Pedersen, Martin Stendel, Nicholas Midzi, Takafira Mduluza, White Soko, Anna-Sofie Stensgaard, Birgitte J Vennervald, Samson Mukaratirwa, Thomas K Kristensen

**Affiliations:** Department of Veterinary Disease Biology, Faculty of Health and Medical Sciences, University of Copenhagen, Dyrlægevej 100, 1870 Frederiksberg C, Denmark; Danish Meteorological Institute, Copenhagen, Denmark; Department of Medical Microbiology, University of Zimbabwe, College of Health Sciences, Harare, Zimbabwe; National Institute of Health Research, Ministry of Health and Child Care, Causeway, Harare, Zimbabwe; Department of Biochemistry, University of Zimbabwe, Harare, Zimbabwe; School of Laboratory Medicine and Medical Sciences, University of KwaZulu-Natal, KwaZulu-Natal, South Africa; Center for Macroecology, Evolution and Climate, Natural History Museum of Denmark, University of Copenhagen, Copenhagen, Denmark; School of Life Sciences, University of KwaZulu-Natal, KwaZulu-Natal, South Africa

**Keywords:** Snail, Species distribution modelling, Climate change, Regional climate models, Schistosomiasis, Fascioliasis

## Abstract

**Background:**

Freshwater snails are intermediate hosts for a number of trematodes of which some are of medical and veterinary importance. The trematodes rely on specific species of snails to complete their life cycle; hence the ecology of the snails is a key element in transmission of the parasites. More than 200 million people are infected with schistosomes of which 95% live in sub-Saharan Africa and many more are living in areas where transmission is on-going. Human infection with the *Fasciola* parasite, usually considered more of veterinary concern, has recently been recognised as a human health problem. Many countries have implemented health programmes to reduce morbidity and prevalence of schistosomiasis, and control programmes to mitigate food-borne fascioliasis. As these programmes are resource demanding, baseline information on disease prevalence and distribution becomes of great importance. Such information can be made available and put into practice through maps depicting spatial distribution of the intermediate snail hosts.

**Methods:**

A biology driven model for the freshwater snails *Bulinus globosus, Biomphalaria pfeifferi* and *Lymnaea natalensis* was used to make predictions of snail habitat suitability by including potential underlying environmental and climatic drivers. The snail observation data originated from a nationwide survey in Zimbabwe and the prediction model was parameterised with a high resolution Regional Climate Model. Georeferenced prevalence data on urinary and intestinal schistosomiasis and fascioliasis was used to calibrate the snail habitat suitability predictions to produce binary maps of snail presence and absence.

**Results:**

Predicted snail habitat suitability across Zimbabwe, as well as the spatial distribution of snails, is reported for three time slices representative for present (1980-1999) and future climate (2046-2065 and 2080-2099).

**Conclusions:**

It is shown from the current study that snail habitat suitability is highly variable in Zimbabwe, with distinct high- and low- suitability areas and that temperature may be the main driving factor. It is concluded that future climate change in Zimbabwe may cause a reduced spatial distribution of suitable habitat of host snails with a probable exception of *Bi. pfeifferi*, the intermediate host for intestinal schistosomiasis that may increase around 2055 before declining towards 2100.

**Electronic supplementary material:**

The online version of this article (doi:10.1186/s13071-014-0536-0) contains supplementary material, which is available to authorized users.

## Background

Schistosomiasis is a major health concern in many parts of the world where an estimated 207 million people are infected and 779 million are at risk of infection [1,2]; and 85% of the people live in countries south of the Sahara. The high prevalence of schistosomiasis in Zimbabwe is well known from a recent national survey carried out in 2010 and 2011 [3]. More than 2.2 million (18%) persons are estimated to be infected with *Schistosoma haematobium*, the cause of urinary schistosomiasis and close to 900,000 (7.2%) with *S. mansoni* causing intestinal schistosomiasis [3] (population numbers based on Tatem *et al.* [4]). Fascioliasis, caused by *Fasciola spp*, primarily known to be of veterinary concern is increasingly recognised to be responsible for morbidity in humans with estimates of up to 17 million human infections [1,5-8]. As in most other parts of the world, the prevalence of human fascioliasis has not been intensively investigated in Zimbabwe, though prevalence of up to 5% has been reported [9,10]. Fascioliasis affects ruminants and prevalence of 90% has been reported in cattle in some areas of Zimbabwe [11].

Parasites and the snail intermediate host are poikilotherms, and their intrinsic rate of development is dependent on temperature, which becomes an indirect predictor of transmission risk, however, other climatic and environmental factors contribute to the delimitation of their spatial distribution. Georeferenced collection-points for snail observations, in combination with environmental predictors, mainly climatic, were used to develop a model for prediction of spatial distribution for each of the three snail species in Zimbabwe, by the use of the Maxent modelling software [12]. The prediction models were parameterised with climate projections using the regional climate model HIRHAM5 [13-16] for periods representative for present-day climate and two future periods.

A recent study from Zimbabwe substantiated how the snail distribution has changed in the 24 year period from 1988 to 2012 and that this may be the consequence of a change in climate [17]. This earlier study focused on short term climatic changes, i.e. year-to-year variability rather than the decadal variability which is investigated in this paper. The question now remains how climate change may affect snail distribution and consequently the impact of schistosomiasis and fascioliasis in the future.

The distribution of the aforementioned parasitic infections is reliant on the presence of their respective intermediate snail host species. Distribution of the snails can thereby provide information on disease distribution though the presence of parasites and exposure are also the determining factors. A unique opportunity of having comprehensive data on schistosomiasis and fascioliasis prevalence from Zimbabwe enabled a translation of the Maxent model output of habitat suitability into a distribution, i.e. delimitation of area of occupation of the snails.

The aim of the current study was to predict the nationwide spatial distribution of three trematode intermediate snail host species: *Bulinus globosus* (Morelet 1866), *Biomphalaria pfeifferi* (Kraus 1848) and *Lymnaea natalensis* (Kraus 1848) for present-day climate, and to forecast the distribution in a future climate, based on a climate change projection model. The overall present and future spatial distribution of potential suitable snail habitat is reported for Zimbabwe and the impact of climate change is discussed. Furthermore, the habitat suitability modelling results are translated into areas of occupation of the three snail species.

## Methods

### Study area and sampling method

Zimbabwe is a landlocked country situated in the southern tropical zone and comprises an area of 390,757 km^2^. Two bio-climatic zones exist, the highveld (1000-1500 m.a.s.l) and lowveld (500-1000 m.a.s.l.), primarily distinguished by high and low rainfall patterns, respectively. The highveld covers most of central Zimbabwe stretching in a southwest-northeast direction and the lowveld covers most of the northwest and southeast. There is a rainy- (Dec- Feb), post-rainy- (Mar -May), cold-dry- (Jun -Aug), and hot-dry (Sep -Nov) season [18-21].

**Figure 1:**
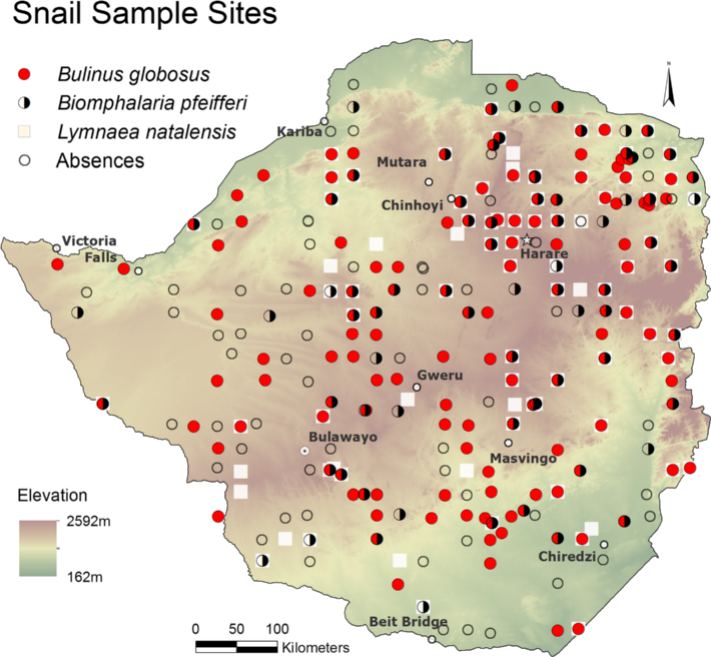
**Snail sample sites and occurrence of three snail species: red circle** ***Bulinus globosus*** **, semi-circle** ***Biomphalaria pfeifferi*** **, open square** ***Lymnaea natalensis*** **, and open circle absences.**

Snail data, used in this analysis, originated from a national snail survey in May and June of 1988, after the rainy season covering all parts of Zimbabwe (Figure 1) [22]. Snail collection methods and equipment were as described by Coulibaly and Madsen [23] and snail identification was done following keys described by Brown and Kristensen [24] by expert malacologists in Harare, Zimbabwe. A total of 18,066 snails representing 19 different species were collected from 364 locations. *Bulinus globosus* were found at 121 locations, *Bi. pfeifferi* at 64, and 74 locations held *L. natalensis*. Sampled habitats were rivers, marshes, pools, dams, springs, and canals at elevations between 221 to 1.595 m.a.s.l. Collection sites were georeferenced by attributing the geographical coordinate of the arithmetic centre of predefined 26.5 km by 26.5 km grid cells.

### Environmental layers

To resolve the topography of Zimbabwe adequately, the highest possible spatial resolution, is necessary. General Circulation Models (GCMs) have typical resolutions on the order of 100 km. The output of the GCM was therefore dynamically downscaled, in this case to a target resolution of 10 km, to drive a regional climate model (RCM). Due to numerical stability reasons; an intermediate downscaling step to 44 km was done. Here we consider only the high resolution 10 km data. The RCM is based on Christensen [13] with subsequent modifications and improvements described in Lucas-Picher *et al.*[25] Rae [14], Mottram [15] and Langen [16]. The modelling domain for the climate projection model covers roughly 12 million km^2^ in eastern Africa between 28°S and 5°N and between 16°E and 52°E. Simulations with such high resolution are computationally very resource and time consuming; therefore we consider here only three time-slices of 20 years each: 1980-1999 (representative for present-day climate) and 2046-2065 and 2080-2099 (representative for future climate), hereafter denoted “1990”, “2055” and “2090”, respectively. The underlying scenario is IPCC SRES A1B [26], which is described by rapid economic growth, a global population of nine billion by 2050 and a balanced emphasis on fossil and non-fossil energy sources. We evaluated temperature, rainfall and humidity for the annual mean and an average for the months March, April and May, due to the fact that these months are important to snail population development in Zimbabwe [27]. Fourteen so-called bioclimatic variables [28] were calculated by the use of DIVA GIS (www.diva-gis.org) following Hijmans *et al*. [29] and Ramírez and Bueno-Cabrera [30] where after they were analysed for collinearity, together with March-April-May averages of precipitation, temperature, and humidity as well as elevation and soil-pH. Based on an exclusion criterion of a collinearity factor of 0.70, variables were excluded from the final model (Additional file 1a, Additional file 2b, and Additional file 3c): the variable with most partners not meeting the criteria were excluded. Average precipitation in the period of March-April-May (MAM) (rr) and average MAM temperature (t2) were chosen above average MAM of relative humidity (rh) due to rh being derived from the two former. The remaining variables used in the model are listed in Table 1.

**Table 1:** **Model test statistics**

	***Bulinus globosus***	***Biomphalaria pfeifferi***	***Lymnaea natalensis***
Suitability range:	0.00 – 0.81	0.00 – 0.88	0.00 – 0.90
AUC test statistics:	0.737	0.771	0.765
MTSPSLT*:	0.45	0.49	0.43
Sensitivity | specificity:	0.64 | 0.53	0.45 | 0.82	0.48 | 0.77
**Variable**	**Variable contribution (%)**		
Temperature March-April-May	**64**	**49**	**70**
Temperature Seasonality	**17**	8	**13**
Precipitation seasonality	4	**18**	NA
Precipitation of wettest month	**11**	NA	5
Precipitation of warmest quarter	NA	NA	4
Precipitation of driest month	2	NA	2
Precipitation of wettest quarter	NA	**16**	NA
Precipitation March, April-May	0	4	5
Temperature of driest quarter	NA	3	NA
Precipitation of driest quarter	NA	0	NA
pH – soil**	2	3	2

Bold: most contributing variables.

*Maximum training sensitivity plus specificity logistic threshold.

**www.isric.org.

### Model implementation

The modelling software, Maxent [12] was used to predict snail habitat suitability from snail presence data according to environment and climatic variables. The output from Maxent was considered as the probability of snail presence expressed as a map layer of habitat suitability on a scale of 0 - 1 for non-suitable and suitable habitat, respectively. The environmental data were loaded into Maxent, covering an area of 10 by 10 arc degrees, encompassing Zimbabwe and parts of neighbouring countries, and had a resolution of 0.1 by 0.1 arc degrees. Maxent was set to sample 10,000 background samples from the environmental variables, during fitting of the distribution model. Collection sites holding one or more specimen of a snail species were introduced by the ascribed coordinates of the collection sites. Sites, from where no specimens of the modelled species were found, were not used in the model. The average of 10 replicate model runs was reported and the model initialisation used random seeds and 10% of the observations were set aside for model testing.

All commonly accepted ecological zones in the modelling domain were present among the snail observation data to comply with Maxent’s constraint to not predict into novel eco-zones [31]. Maxent provides a number of arithmetic products of the predictors denoted as “feature classes” which, in this study, were limited to “linear” and “quadratic”, omitting “product”, “threshold”, and “hinge”, due to the non-intuitive, function of these features, in terms of snail biology, and due to the non-linear response of the species to some of the environmental variables, following the recommendations of Merow [31]. Area Under the receiver operator characteristic Curves (AUC) of the test data are reported as an expression of model performance as suggested by Liu [32], and is supported by measures of sensitivity and specificity following recommendations by Hu and Jiang [33]. A build-in, Jack-knife procedure was used to quantify the explanatory power of each environmental variable.

The 1990 climate projection data were used to fit the snail habitat suitability prediction model and subsequently parameterised with climate projection data for 2055 and 2090, to produce a climate change impact prediction.

## Results

**Figure 2:**
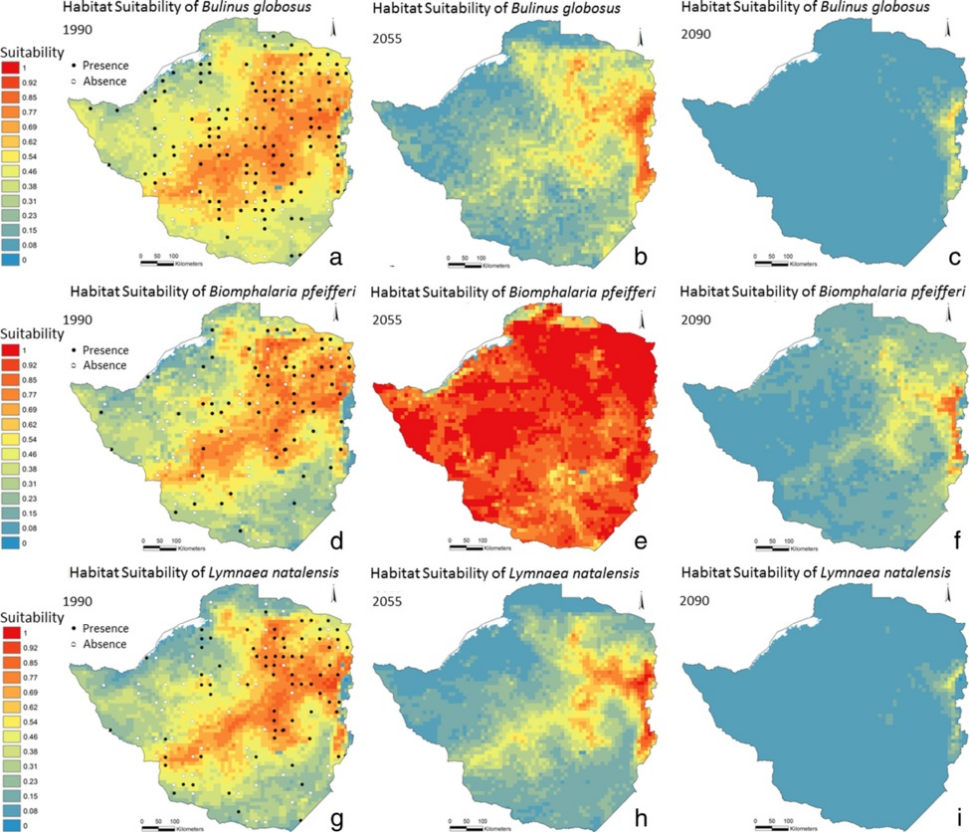
**Predicted relative habitat suitability for three species of snails in three time periods.** *Bulinus globosus* **(a, b and c)**, *Biomphalaria pfeifferi* **(d, e and f)**, and *Lymnaea natalensis* **(g, h and i)**. Habitat suitability increases from blue via yellow to red. Left column: present-day climate (1980-1999), mid-column: near-future climate (2046-2065), right column: end century climate (2080-2099).

**Figure 3:**
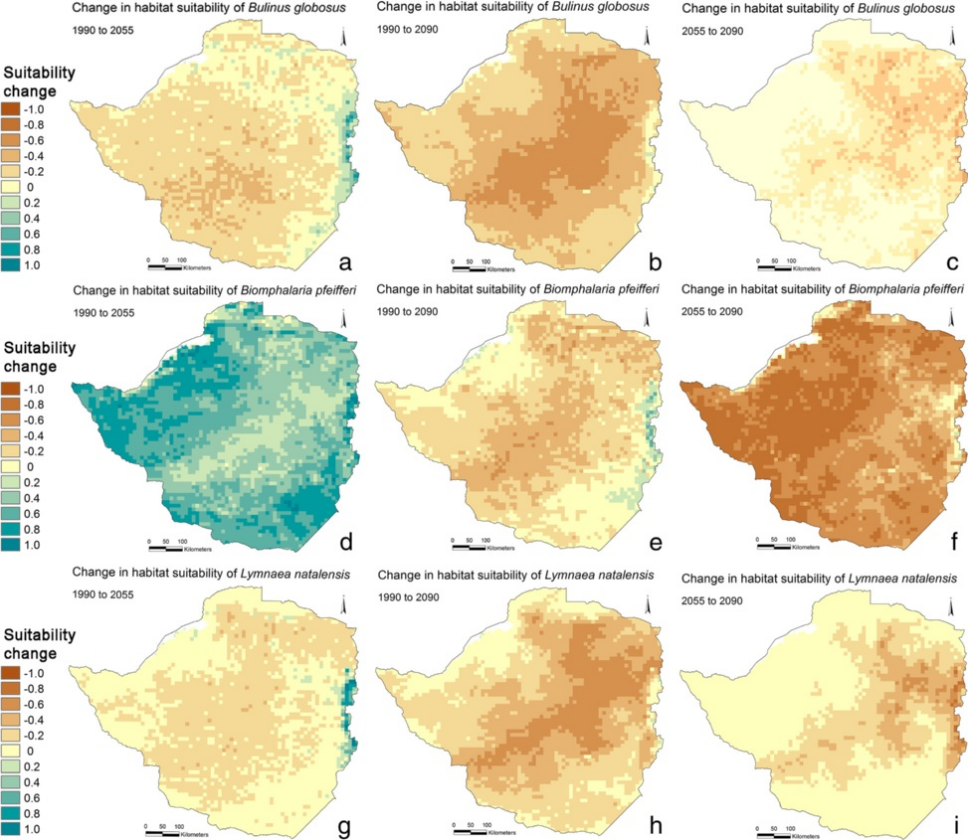
**Predicted changes in relative habitat suitability index for three species of snails in three time periods (a-i).** Blue colours indicate an increase in habitat suitability, yellow no change, and brown a decrease in suitability.

Predicted snail habitat suitability across Zimbabwe for the three snail species in three different periods are presented in Figure 2. Results for *B. globosus* show high suitability in the highveld for 1990 (Figure 2a), whereas areas to the southeast and northwest are predicted to be less suitable. The predictions for 2055 and 2090 (Figure 2b and c) illustrate that fewer locations are predicted to be suitable compared to that of 1990, with only few locations of relatively high suitability outside the eastern highlands of Zimbabwe, by 2090. The most significant drop in suitability index in the period from 1990 to 2055 is observed in the central and southern part of Zimbabwe (Figure 3a), and all parts of Zimbabwe, including the highveld, is reduced significantly between 1990 and 2090 (Figure 3b). Furthermore, there is a falling trend in habitat suitability in both of the two interim periods of 1990 to 2055 and 2055 to 2090 (Figure 3a and Figure 3c, respectively).

The prediction of *Bi. pfeifferi* is depicted in Figure 2d–f. The highveld and eastern highlands constituted the most suitable habitat in 1990 with a more distinct gradient between high- and lowveld compared to that of *B. globosus*. All parts of Zimbabwe are predicted to be highly suitable by 2055 (Figure 2e) forming the basis for increased transmission risk of intestinal schistosomiasis, but with a significant reduction toward the end of the century; however, areas with medium suitability are still present in the central highveld in 2090 (Figure 2f). *Biomphalaria pfeifferi* experience an increasingly favourable climate towards 2055 (Figure 3d) and, like *B. globosus*, a reduction towards the late century (Figure 3f). It is noteworthy that *Bi. pfeifferi* does not experience a linear habitat suitability reduction throughout the modelling period.

Suitable habitats for *L. natalensis* (Figure 2g) are predicted for the highveld and in the southern part of Zimbabwe for 1990. Furthermore, areas with low values for suitable habitats are present to the northwest and southeast. It is predicted that the distribution of suitable habitats are reduced in 2055 though relatively suitable habitats are present in large parts of the former core areas (Figure 2h). By 2090, most of the country is absent of suitable habitat, where only the very central highveld and the eastern highlands are predicted to be relatively suitable habitat (Figure 2i). The reduction follows a steady gradient throughout the modelling period (Figure 3g and Figure 3i).

Model test statistics to establish model performance for predicting habitat suitability, and the accuracy of snail occurrence predictability are reported in Table 1 in the form of AUC, and measures of sensitivity and specificity. AUC values of 0.737, 0.771, and 0.765 (*B. globosus, Bi. pfeifferi, and L. natalensis*, respectively) indicate acceptable modelling performance whereas sensitivity scores of 0.45 and 0.48 for *Bi. pfeifferi* and *L. natalensis*, respectively, indicate poor ability to predict where snails are present. The model for *B. globosus* is to some extent better at predicting true presence with a sensitivity score of 0.64. The ability of the model to predict areas where snails are absent is fairly good for *Bi. pfeifferi* and *L. natalensis* with specificity scores of 0.82, and 0.77, respectively, whereas the score for *B. globosus* is low (0.53).

**Figure 4:**
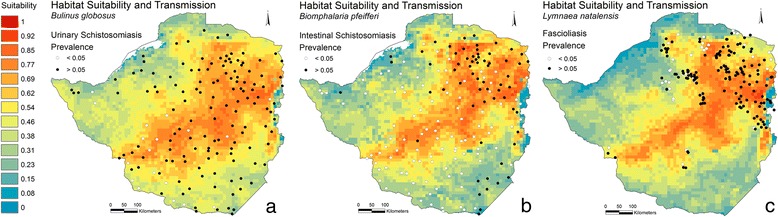
**Predicted snail habitat suitability in 1990 for three snail species.** *Bulinus globosus* **(a)**, *Biomphalaria pfeifferi* **(b)** and *Lymnaea natalensis* **(c)** with an overlay of prevalence survey schools (human schistosomiasis - map a and b) and dip tanks (veterinary fascioliasis - map c). ◯ no transmission (prevalence <5%), ● on-going transmission (prevalence >5%).

**Figure 5:**
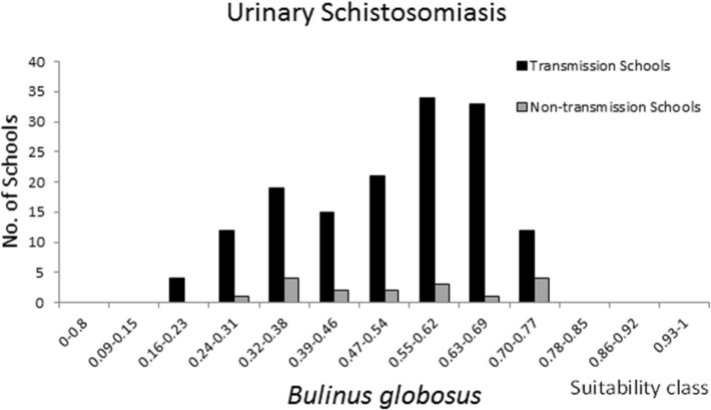
**Frequency distribution of schools with on-going, and no transmission of** ***Schistosoma haematobium*** **in 14 classes of** ***Bulinus globosus*** **habitat suitability.**

**Figure 6:**
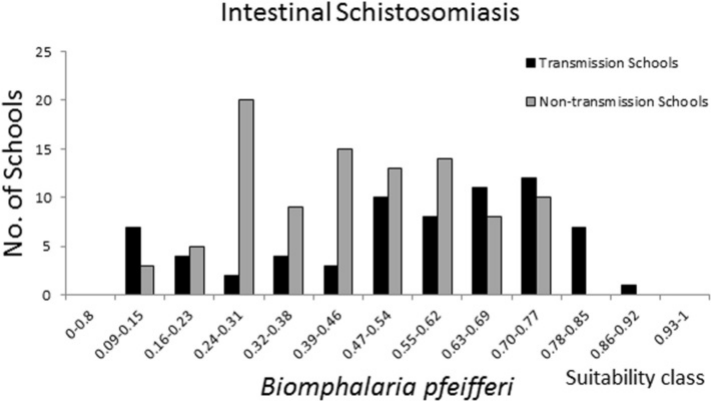
**Frequency distribution of schools with on-going, and no transmission of** ***Schistosoma mansoni*** **in 14 classes of** ***Biomphalaria pfeifferi*** **habitat suitability.**

**Figure 7:**
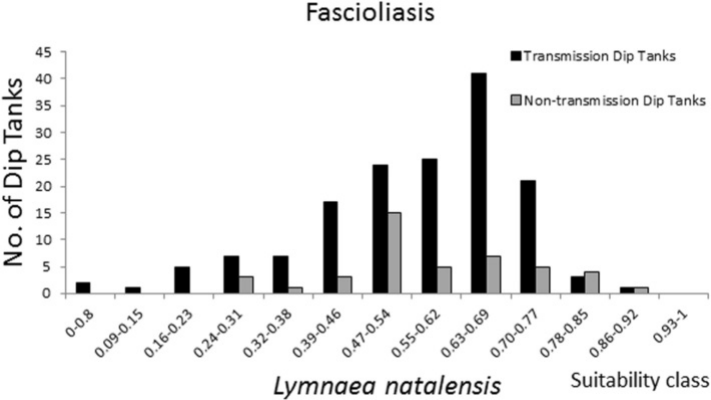
**Frequency distribution of diptanks with detected** ***fasciola gigantica*** **infection in cattle in 14 classes of** ***Lymnaea natalensis*** **habitat suitability.**

Maxent provides a probability of habitat suitability; although, this does not inform the actual distribution of snails. Lending information from disease data can help to identify an approximated index value of habitat suitability that delimits the snail distribution. Raw prevalence data on schistosomiasis prevalence among school-aged children from a national survey conducted in 1981 [34], was provided by the National Institute of Health Research, Zimbabwe. Prevalence of the infection status was determined using microscopic examination of urine and faeces samples as described by Taylor and Makura [34] from randomly selected children at 157 primary schools representing all regions of Zimbabwe. Fascioliasis prevalence data in cattle were obtained by microscopy of faeces sampled at dip tank sites and provided by the Central Veterinary Laboratory, Zimbabwe. Sampling was conducted in the period of 1989 to 1993, January-December, up to 1.747 m.a.s.l. at 197 locations, mainly in the north-eastern highveld. Overlaying the prevalence data on the suitability maps, reveals the locations of disease transmission (here defined as schools or dip tanks with prevalence above 5%) and the respective suitability index value (Figure 4). Inspecting these prevalence and suitability classes on a frequency distribution plot (Figures 5, 6, and 7) allow visual inspection of infection-status as a function of suitability and can help to estimate the suitability index values where snail populations are likely to be viable. From the plot for *B. globosus* (Figure 5) it can be seen that a high number of transmission-schools are present in classes with high suitability scores. At the same time it is observed that there are many schools with no transmission in the lower suitability classes. Given the uncertainties in the prediction model, one may conclude that the snail populations are viable at index values above approximately 0.5. Many schools with on-going transmission are present, even in areas with low suitability index for *Bi. pfeifferi* (Figure 6) and this may be interpreted as *Bi. pfeifferi* being able to exist in areas with relatively low suitability index values (>0.3). For *L. natalensis*, many transmission sites are found in suitability index classes of above 0.47, and dip tanks where there is no transmission are present in low index classes (Figure 7). On this basis, a delimitation of > 0.5 may be suggested.

An entirely statistical approach to delimitate suitable versus unsuitable habitat can be used by making use of a statistical output from Maxent: the “maximum test sensitivity plus specificity logistic threshold”. These statistics state that the threshold for viable snail populations should be found at habitat suitability index classes of 0.45, 0.49, and 0.43 for *B. globosus, Bi. pfeifferi*, and *L. natalensis*, respectively (Table 1). Binary maps based on these thresholds are presented in Additional file 4.

**Figure 8:**
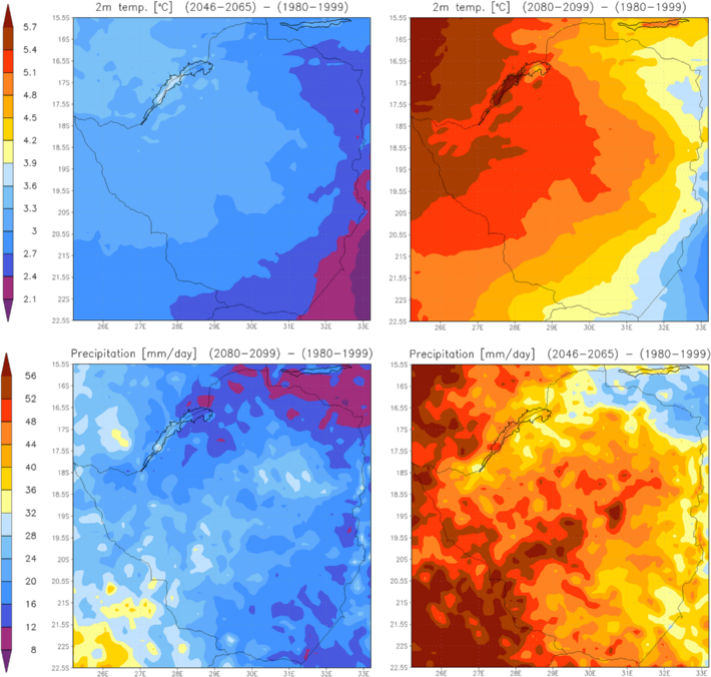
**Projected changes of temperature (K) and precipitation (expressed in % of present-day value).** First column: change towards mid-century, second column: change towards end century.

Figure 8 shows the projected temperature and precipitation changes for the period 2080-2099 with respect to 1980-1999 for the annual average. Generally, future conditions can be described as warmer as and drier than at present. Temperatures are projected to increase in all parts of Zimbabwe and most severely in the northwest with maximum values above 5 K, near the Zambian border. Annual rainfall is projected to decrease considerably; least in the southwest (roughly 1/3 of present-day values) and most in the northeast, where only about 15% of present-day precipitation is projected for the annual mean. The post-rainy season (March to May) is projected to be much drier, with generally less than 10% of present-day values (Additional file 5).

## Discussion

In this study, it is shown by predictions of snail habitat suitability for *B. globosus, Bi. pfeifferi* and *L. natalensis*, that there is a distinct gradient of suitability across Zimbabwe. The three species share large areas with high suitability but also have unique “hot-spots”. Changes of spatial distribution in the future climate of 2055 and 2090 are apparent with a trend towards more locations with unsuitable habitats; though suitable habitats are still present (Figure 2). The predicted distribution of *Bi. pfeifferi* in 2055 indicates a substantial increase in habitat suitability. If this expansion is ascribed to the 3.1°C increase in the period averages, as it is observed in the temperature data for March, April, and May (Additional file 5), it can be concluded that the *Bi. pfeifferi* snail tolerates higher temperatures than the other two species. Even so, the temperature becomes above optimal at the end of the century. *Biomphalaria pfeifferi* is also the species that finds most suitable habitats in 2090 and in fact have a substantial area of distribution (Additional file 4f). The area of occupation, as opposed to habitat suitability, for the three snail species is estimated by evaluating site specific parasite transmission status in relation to habitat suitability index, and by test statistic. This information may suggest that snail populations are viable at approximately a suitability index of >0.4 with some variation depending on species, method, or purpose. It should be noted that defining a threshold can be controversial and may depend on the purpose. Conservationists, for example, may argue for a less conservative delamination (lower threshold) in questions regarding habitat protections. It may seem surprising that there are schools and dip tanks where transmission is on-going in almost all of the index classes, even classes with suitability index below that of the suggested 0.4. This may be explained by imported cases carrying transmission from elsewhere where habitat suitability is in fact high, a too coarse resolution of the model output or simply that snail populations are viable even at the lowest suitability index classes. The transmission-positive schools in low index classes along the shores of Lake Kariba may be an artefact of the model perhaps not “catching” the truly suitable habitats (the model may misclassify these areas and assign erroneously low suitability index). In addition, it is shown that many schools where transmission was not occurring are present in areas with highly suitable habitats which may be explained by possible on-going local treatment campaigns and/or by prioritisation of schistosomiasis survey efforts in areas otherwise known to have low incidence of schistosomiasis. *Fasciola* transmission-positive dip tanks in areas with a suitability index below 0.4 may be explained by dip tanks having a large catchment area i.e. cattle have been infected in adjacent high-index areas. In fact, the positive dip tanks at low suitability class locations are all found in areas close to the line of delimitation (Additional file 4g). Finally, the fact that the prevalence data of both schistosomiasis and fascioliasis, and snail observation data do not overlap in time, will inevitably lead to some deviations.

Variable-contribution reported in Table 1 informs about what factors might be driving the distribution model. The average temperature of March-May is by far the most contributing factor for the three snail species (49% to 70%) indicating that temperature may be the main driver for the distribution. All other variables have a contribution of 18% or less, and temperature (seasonality), again, has a higher degree of contribution together with two datasets on precipitation (seasonality and precipitation in the wettest month).

The AUC statistics indicate an “acceptable” model performance [35] but low sensitivity for *Bi. pfeifferi* and *L. natalensis*, implying that the model is less capable of predicting where these species are present, whereas more confidence can be put into the model’s ability to predict where snails are not likely to be found. For *B. globosus* the situation is the opposite with better performance at predicting true positives as opposed to true negatives.

The quality of input data greatly influences the performance of any model. Snail occurrence data used in this study, are “plenty” for Maxent to characterise the environment at sampling sites [36]. Sampling bias greatly influences reliability of model output. We do not have control of sampling procedure but know that many types of habitats have been sampled e.g. ponds, rivers etc. and in most parts of Zimbabwe. Additionally, we find many absence observations in the original dataset, suggesting that collection sites were not chosen after where specific species were expected to be present. In fact we see that sampling success rates are similar to that of the authors’ own study [17] where special attention was paid to sampling bias. Environmental data are of satisfactory resolution though more variables like e.g. NDVI and Growing Degree Days may have contributed to model reliability.

Compared to other combinations of GCMs and RCMs, the present study yields very dry future climate, even though the precipitation changes in the driving GCM (not shown) are much smaller. This somewhat counterintuitive behaviour can be explained with changes in soil moisture. In the HIRHAM RCM, soil moisture dries faster than in the GCM, thus leading to a further increase in temperature and less precipitation. There are some indications [37,38] for a decrease of precipitation during March-May, but the overall model spread is rather large and the mechanisms are not well understood [39].

The climate model data used in this study is downscaled from relatively low-resolution into high resolution regional fields, but the regional model can evolve freely apart from the forcing data moving into or out of the RCM domain from the driving model. Since the driving data is from a model rather than observations, individual events cannot be compared directly; however, in a statistical sense such a comparison is possible. Information on in-year weather extremes could therefore have been taken into account in this study but due to the data implementation, using 20 year averages, such weather events were not present in the data. Extreme events, such as floods, dry spells, and heat waves would most likely cause an even further reduction in the snail habitat suitability, as snails cannot exist in water at higher velocities than 0.3 m/s [40] and they can only survive dry-spells for a limited period of time [41,42].

The temperature is expressed as ambient temperature at 2 m above ground as opposed to temperature in the habitat water. The correlation between ambient- and water temperature may change between locations and the relationship may change in changed climate conditions [43].

Describing alkalinity (pH) of habitat water and its relation to snail biology has proved complicated. Diurnal variation, of photosynthesis in the water, faecal contamination, and upstream soil pH influence the snails in a non-straightforward manner [40,44,45] but the models still include the pH dataset as a predictor. Furthermore, the pH dataset used here [46] is based on pH in soil water and it is possible that geophysical characteristics are the underlying driver.

Some flaws in the data and modelling implementation can compromise conclusions on habitat suitability, distribution and impact of climate change. Global Positioning Systems (GPS) were not readily accessible in 1988 wherefore sampling locations were simply designated the arithmetic centre of a predefined grid of 26.5 km by 26.5 km. The consequence is that the collection sites and the environmental variables (10 km by 10 km resolution) are misaligned at some locations. There is a number of reasons why this is not considered to conflict with the conclusions of the modelling results: i) the variables most often have similar values in neighbouring cells, ii) variables are averages taken over a 20 year period, iii) and in some cases, averages over three months.

## Conclusions

The presence of intermediate host snails is pivotal for disease transmission but at the same time it is not the only element in the parasite life cycle. Climatic variables and the geophysical environment also influence directly on the schistosome and *Fasciola* parasites’ free living life stages i.e. egg, miracidia, cercaria, and metacercaria (*Fasciola*). Thus, when discussing snail habitat suitability as predictor for schistosomiasis and fascioliasis, modelling of cercaria survival could be included to give an advantage such as exemplified by Stensgaard [47] and Valencia-Lopez [48], where development rate of the cercaria in relation to temperature was included.

In the present study the models based on snail presence data and climatic/environmental input data for two different time periods suggested that snail populations will experience less favourable conditions in Zimbabwe in the future, except for *Bi. pfeifferi* in mid-century. Some populations within Zimbabwe are already at the edge of their range of occupation, wherefore some populations are likely to disappear and consequently parts of Zimbabwe could become free of transmission of schistosomiasis and fascioliasis, though it may be speculated that a series of more favourable years in a generally unfavourable climate period can lead to re-establishment of snail population and subsequently transmission. An important factor would be the rate of reestablishment of snail populations, and parasite re-introduction. Snails are known to spread fast by eggs being transported by aquatic birds on feet or in plumage [49-52] and parasites can be introduced rapidly by infected human and animals. C Appleton and H Madsen [53] describe the re-emergence of schistosomiasis in a community in South Africa where it is indicated that the reintroduction correlated with climate fluctuations. In-depth studies on re-emergence of disease, including timelines and climate, based on the biological studies of snails and parasites and change in the environment can provide knowledge on the challenges in the future.

Finally, climate change may drive schistosomiasis and fascioliasis towards elimination in Zimbabwe in the far future of 2090, although other factors such as land-use changes, transmission awareness and interventions may play an important role on the distribution and may in fact overrule that of climate.

## Additional files

Additional file 1:
**Collinearity analysis for environmental and climatic factors for** ***Bulinus globosus.*** rr = average precipitation in the period March-April-May. t2 = average temperature in the period March-April-May. rh = average relative humidity in the period March-April-May. Elevation = elevation. pH = pH. BIO1 = Annual Mean Temperature. BIO4 = Temperature Seasonality (standard deviation *100). BIO8 = Mean Temperature of Wettest Quarter. BIO9 = Mean Temperature of Driest Quarter. BIO10 = Mean Temperature of Warmest Quarter. BIO11 = Mean Temperature of Coldest Quarter. BIO12 = Annual Precipitation. BIO13 = Precipitation of Wettest Month. BIO14 = Precipitation of Driest Month. BIO15 = Precipitation Seasonality (Coefficient of Variation). BIO16 = Precipitation of Wettest Quarter. BIO17 = Precipitation of Driest Quarter. BIO18 = Precipitation of Warmest Quarter. BIO19 = Precipitation of Coldest Quarter. Additional file 2:
**Collinearity analysis for environmental and climatic factors for** ***Biomphalaria pfeifferi.*** rr = average precipitation in the period March-April-May. t2 = average temperature in the period March-April-May. rh = average relative humidity in the period March-April-May. Elevation = elevation. pH = pH. BIO1 = Annual Mean Temperature. BIO4 = Temperature Seasonality (standard deviation *100). BIO8 = Mean Temperature of Wettest Quarter. BIO9 = Mean Temperature of Driest Quarter. BIO10 = Mean Temperature of Warmest Quarter. BIO11 = Mean Temperature of Coldest Quarter. BIO12 = Annual Precipitation. BIO13 = Precipitation of Wettest Month. BIO14 = Precipitation of Driest Month. BIO15 = Precipitation Seasonality (Coefficient of Variation). BIO16 = Precipitation of Wettest Quarter. BIO17 = Precipitation of Driest Quarter. BIO18 = Precipitation of Warmest Quarter. BIO19 = Precipitation of Coldest Quarter. Additional file 3:
**Collinearity analysis for environmental and climatic factors for** ***Lymnaea natalensis***. rr = average precipitation in the period March-April-May. t2 = average temperature in the period March-April-May. rh = average relative humidity in the period March-April-May. Elevation = elevation. pH = pH. BIO1 = Annual Mean Temperature. BIO4 = Temperature Seasonality (standard deviation *100). BIO8 = Mean Temperature of Wettest Quarter. BIO9 = Mean Temperature of Driest Quarter. BIO10 = Mean Temperature of Warmest Quarter. BIO11 = Mean Temperature of Coldest Quarter. BIO12 = Annual Precipitation. BIO13 = Precipitation of Wettest Month. BIO14 = Precipitation of Driest Month. BIO15 = Precipitation Seasonality (Coefficient of Variation). BIO16 = Precipitation of Wettest Quarter. BIO17 = Precipitation of Driest Quarter. BIO18 = Precipitation of Warmest Quarter. BIO19 = Precipitation of Coldest Quarter. Additional file 4: Figure 2a-i. Binary maps of presence (red) and absence (blue) for three snail species at three time periods. Additional file 5:
**Precipitation and temperature (March-April-May averages) for three time-slices: 1980-1999, 2046-2065, and 2080-2099.**
