# Supplementary figures and images for: Modelling climate change impact on the spatial distribution of fresh water snails hosting trematodes in Zimbabwe
Source: Parasit Vectors. 2014 Dec 12;7:536. doi: 10.1186/s13071-014-0536-0 (PMC4299310; doi:10.1186/s13071-014-0536-0)

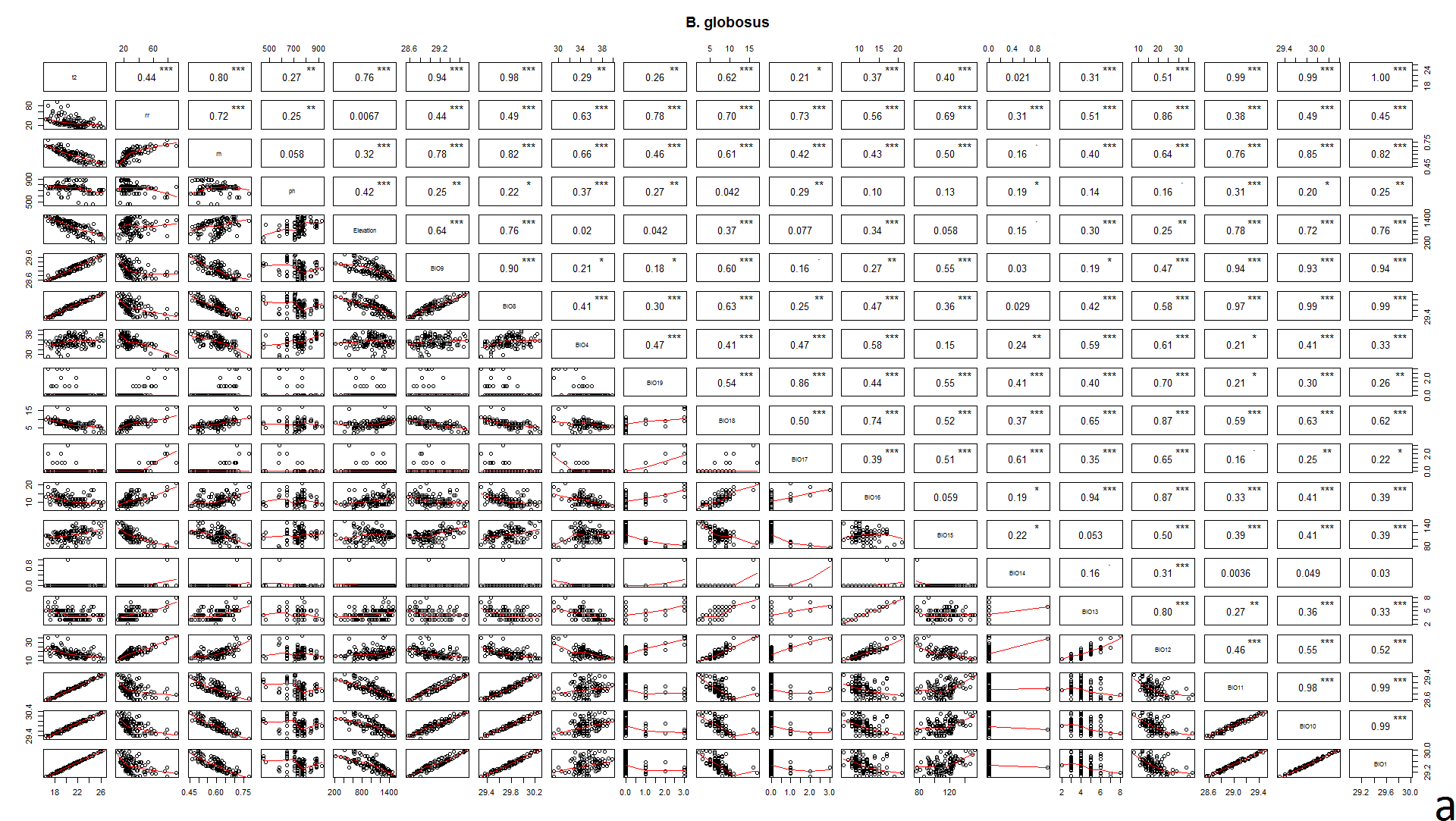

Supplement: Additional file 1: — Collinearity analysis for environmental and climatic factors for Bulinus globosus. rr = average precipitation in the period March-April-May. t2 = average temperature in the period March-April-May. rh = average relative humidity in the period March-April-May. Elevation = elevation. pH = pH. BIO1 = Annual Mean Temperature. BIO4 = Temperature Seasonality (standard deviation *100). BIO8 = Mean Temperature of Wettest Quarter. BIO9 = Mean Temperature of Driest Quarter. BIO10 = Mean Temperature of Warmest Quarter. BIO11 = Mean Temperature of Coldest Quarter. BIO12 = Annual Precipitation. BIO13 = Precipitation of Wettest Month. BIO14 = Precipitation of Driest Month. BIO15 = Precipitation Seasonality (Coefficient of Variation). BIO16 = Precipitation of Wettest Quarter. BIO17 = Precipitation of Driest Quarter. BIO18 = Precipitation of Warmest Quarter. BIO19 = Precipitation of Coldest Quarter. [file 13071_2014_536_MOESM1_ESM.tiff]

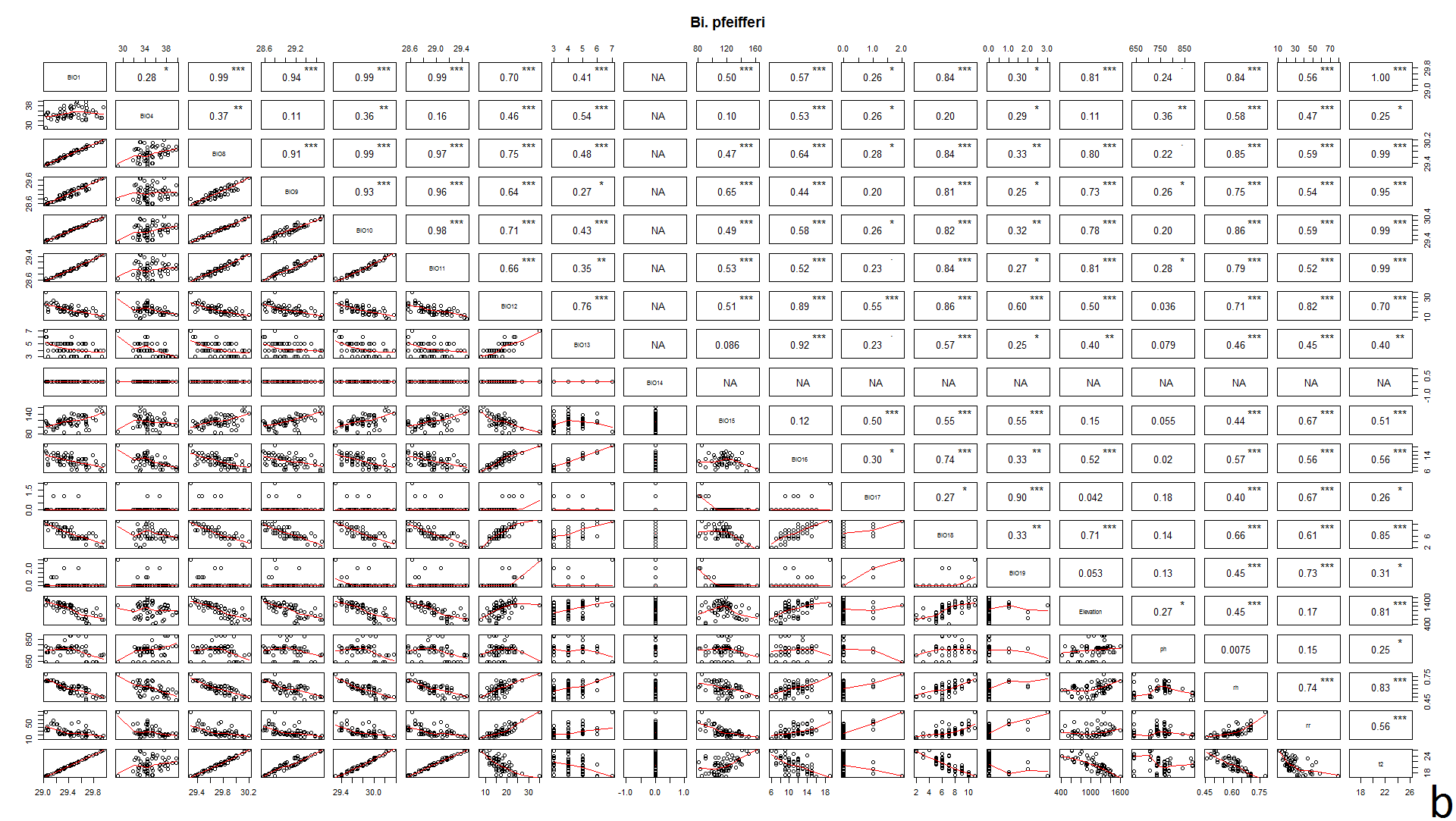

Supplement: Additional file 2: — Collinearity analysis for environmental and climatic factors for Biomphalaria pfeifferi. rr = average precipitation in the period March-April-May. t2 = average temperature in the period March-April-May. rh = average relative humidity in the period March-April-May. Elevation = elevation. pH = pH. BIO1 = Annual Mean Temperature. BIO4 = Temperature Seasonality (standard deviation *100). BIO8 = Mean Temperature of Wettest Quarter. BIO9 = Mean Temperature of Driest Quarter. BIO10 = Mean Temperature of Warmest Quarter. BIO11 = Mean Temperature of Coldest Quarter. BIO12 = Annual Precipitation. BIO13 = Precipitation of Wettest Month. BIO14 = Precipitation of Driest Month. BIO15 = Precipitation Seasonality (Coefficient of Variation). BIO16 = Precipitation of Wettest Quarter. BIO17 = Precipitation of Driest Quarter. BIO18 = Precipitation of Warmest Quarter. BIO19 = Precipitation of Coldest Quarter. [file 13071_2014_536_MOESM2_ESM.tiff]

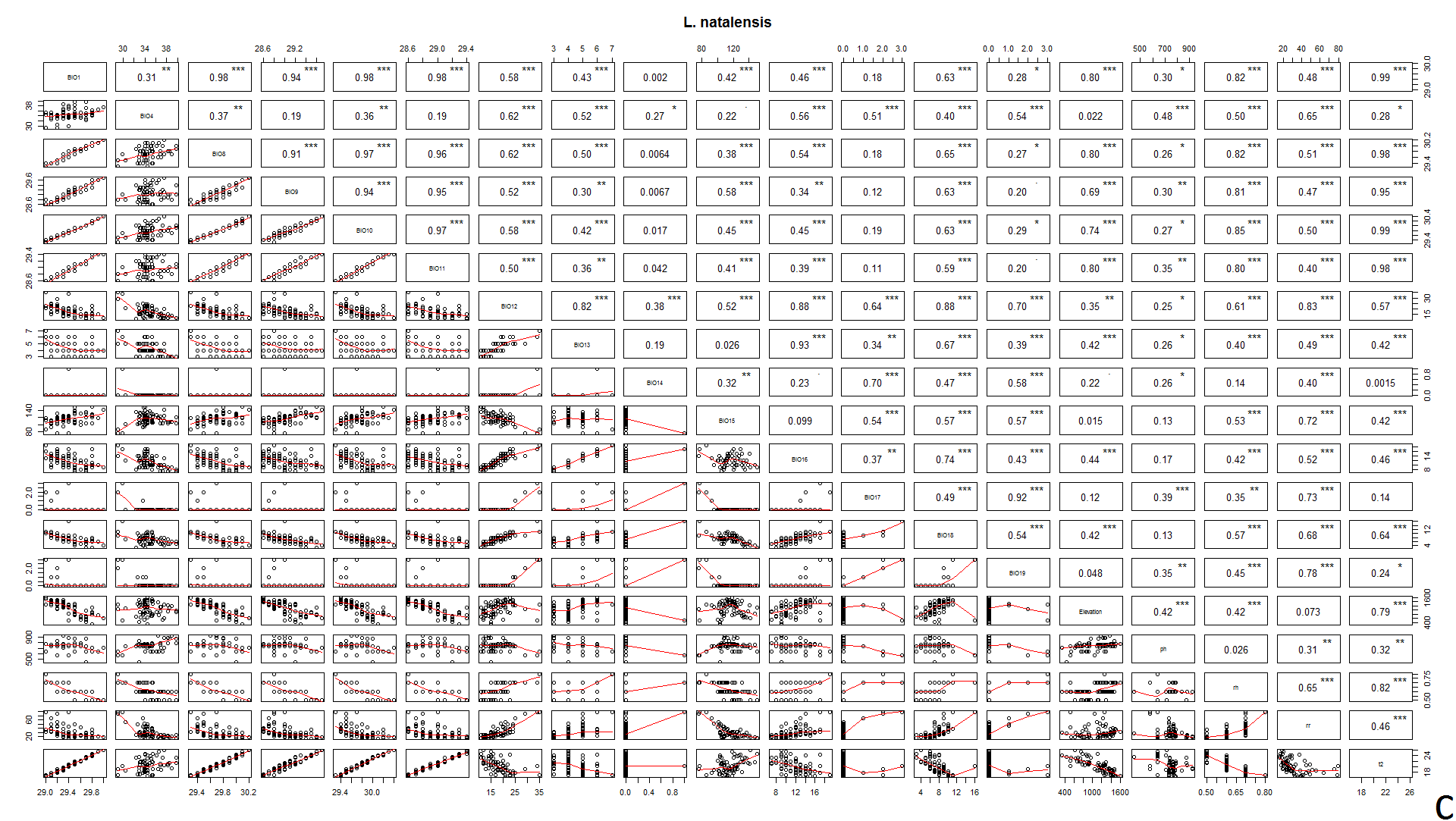

Supplement: Additional file 3: — Collinearity analysis for environmental and climatic factors for Lymnaea natalensis. rr = average precipitation in the period March-April-May. t2 = average temperature in the period March-April-May. rh = average relative humidity in the period March-April-May. Elevation = elevation. pH = pH. BIO1 = Annual Mean Temperature. BIO4 = Temperature Seasonality (standard deviation *100). BIO8 = Mean Temperature of Wettest Quarter. BIO9 = Mean Temperature of Driest Quarter. BIO10 = Mean Temperature of Warmest Quarter. BIO11 = Mean Temperature of Coldest Quarter. BIO12 = Annual Precipitation. BIO13 = Precipitation of Wettest Month. BIO14 = Precipitation of Driest Month. BIO15 = Precipitation Seasonality (Coefficient of Variation). BIO16 = Precipitation of Wettest Quarter. BIO17 = Precipitation of Driest Quarter. BIO18 = Precipitation of Warmest Quarter. BIO19 = Precipitation of Coldest Quarter. [file 13071_2014_536_MOESM3_ESM.tiff]

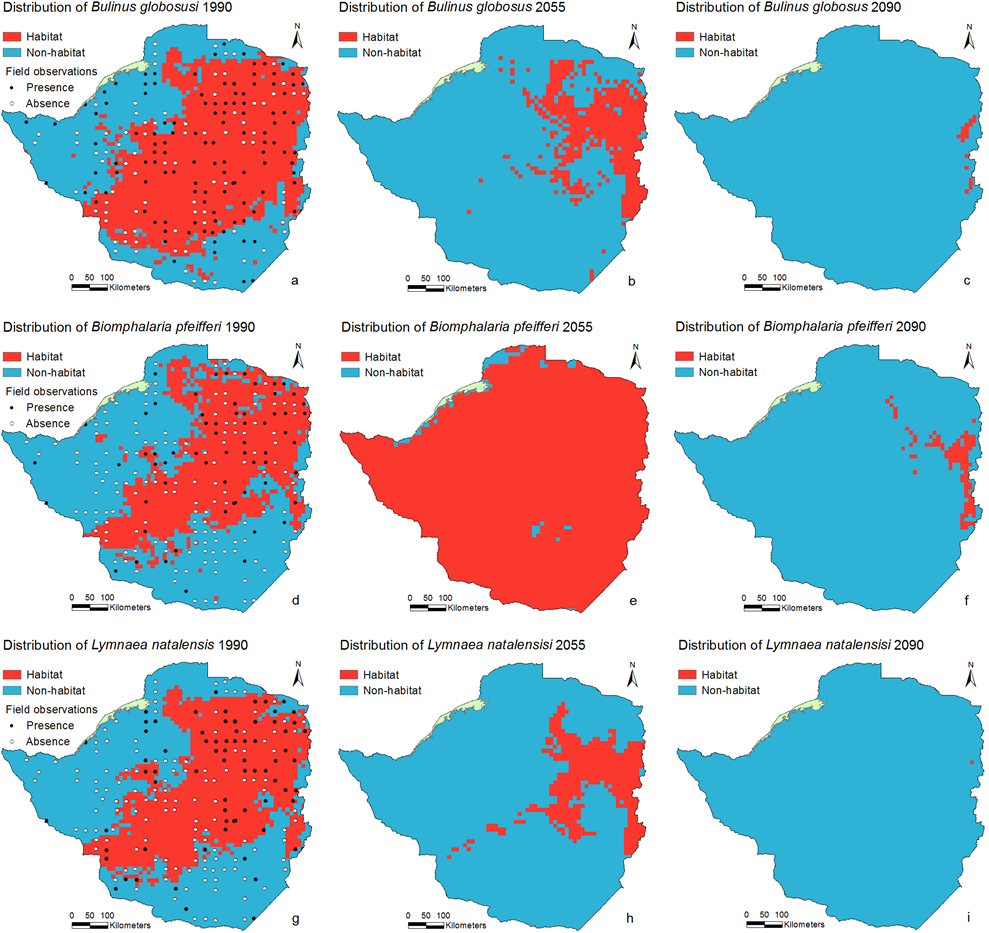

Supplement: Additional file 4: Figure 2a-i. — Binary maps of presence (red) and absence (blue) for three snail species at three time periods. [file 13071_2014_536_MOESM4_ESM.tiff]

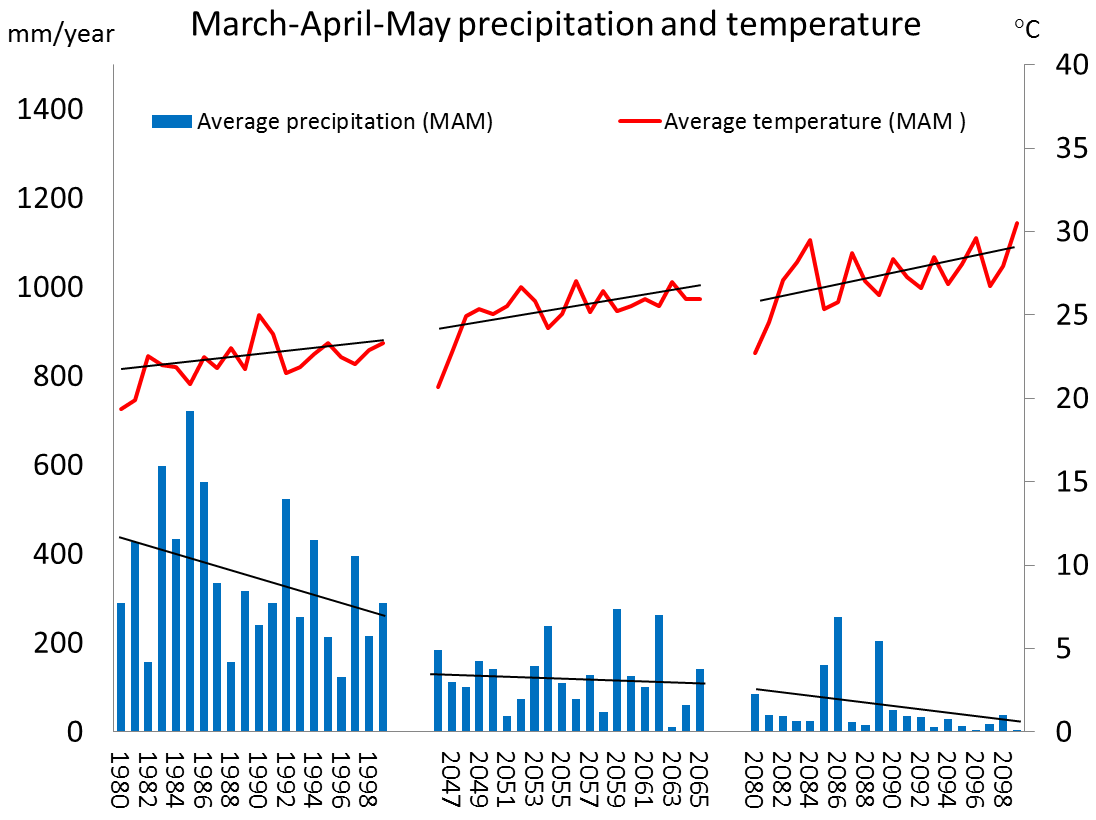

Supplement: Additional file 5: — Precipitation and temperature (March-April-May averages) for three time-slices: 1980-1999, 2046-2065, and 2080-2099. [file 13071_2014_536_MOESM5_ESM.tiff]
